# The Macroecology of Airborne Pollen in Australian and New Zealand Urban Areas

**DOI:** 10.1371/journal.pone.0097925

**Published:** 2014-05-29

**Authors:** Simon G. Haberle, David M. J. S. Bowman, Rewi M. Newnham, Fay H. Johnston, Paul J. Beggs, Jeroen Buters, Bradley Campbell, Bircan Erbas, Ian Godwin, Brett J. Green, Alfredo Huete, Alison K. Jaggard, Danielle Medek, Frank Murray, Ed Newbigin, Michel Thibaudon, Don Vicendese, Grant J. Williamson, Janet M. Davies

**Affiliations:** 1 Department of Archaeology and Natural History, College of Asia and the Pacific, Australian National University, Canberra, Australian Capital Territory, Australia; 2 School of Plant Science, University of Tasmania, Hobart, Tasmania, Australia; 3 School of Geography, Environment and Earth Sciences, Victoria University of Wellington, Wellington, New Zealand; 4 Menzies Research Institute Tasmania, University of Tasmania, Hobart, Tasmania, Australia; 5 Department of Environment and Geography, Faculty of Science, Macquarie University, Sydney, New South Wales, Australia; 6 Center for Allergy and Environment, Technical University of Munich, Munich, Germany; 7 School of Agriculture and Food Science, The University of Queensland, St Lucia, Queensland, Australia; 8 School of Public Health and Human Biosciences, La Trobe University, Bundoora, Victoria, Australia; 9 Allergy and Clinical Immunology Branch, Health Effects Laboratory Division, National Institute for Occupational Safety and Health, Centre for Disease Control and Prevention, Morgantown, West Virginia, United States of America; 10 Plant Functional Biology and Climate Change, University of Technology, Sydney, New South Wales, Australia; 11 School of Medicine, Australian National University, Canberra, Australian Capital Territory, Australia; 12 School of Environmental Science, Murdoch University, Murdoch, Western Australia, Australia; 13 School of Botany, University of Melbourne, Melbourne, Victoria, Australia; 14 European Aerobiology Society, Réseau National de Surveillance Aérobiologique, Lyon, Rhône-Alpes, France; 15 Lung and Allergy Research Centre, School of Medicine, and Translational Research Institute, The University of Queensland, Woolloongabba, Queensland, Australia; The Ohio State University, United States of America

## Abstract

The composition and relative abundance of airborne pollen in urban areas of Australia and New Zealand are strongly influenced by geographical location, climate and land use. There is mounting evidence that the diversity and quality of airborne pollen is substantially modified by climate change and land-use yet there are insufficient data to project the future nature of these changes. Our study highlights the need for long-term aerobiological monitoring in Australian and New Zealand urban areas in a systematic, standardised, and sustained way, and provides a framework for targeting the most clinically significant taxa in terms of abundance, allergenic effects and public health burden.

## Introduction

Pollen is the microscopic haploid stage of the plant life cycle. Pollen grains from different plant species have a remarkable diversity of shapes, sizes, and biochemical compositions. The identification of fossilised pollen in sedimentary sequences (palynology) has been pivotal in illuminating past environmental changes caused by, or associated with, climate change, human impacts and natural disturbances such as fires and tectonic activity. This technique is dependent on the assumption that deposited pollen provides a faithful representation of the vegetation patterns surrounding sedimentary traps. Palynologists test this assumption by collecting pollen from the ground surface within or between typical vegetation types or by sampling pollen in the atmosphere (e.g., D'Costa and Kershaw [1]; Wilmshurst and McGlone [2]; Fletcher and Thomas [3]; Tng *et al*. [4]). These investigations of ‘modern pollen rain’ have focused on wilderness or rural areas, to provide a basis for reconstructing historic biogeographic patterns. How pollen rain varies geographically within urban environments in Australia and New Zealand, where over 85% of the population live in Australia and New Zealand, remains poorly characterised (see Tng *et al*. [4] for an exception).

Parallel studies have produced substantial time series of airborne pollen data for urban locations to underpin investigations of seasonal allergic reaction such as rhinitis or asthma in humans. These large data sets have enabled the development of predictive models between meteorological variables and the concentration of pollen from specific allergenic pollen taxa such as grass and birch (e.g., Schäppi *et al*. [5]; Emberlin *et al*. [6]; Rodríguez-Rajo *et al*. [7]; Sofiev *et al*. [8]). In addition, in some urban areas, particularly in Europe, the aerobiological datasets now span several decades and are beginning to provide insights into biogeographic variation in landscape phenological patterns, and how these patterns respond to current climate change [9]–[12].

In contrast to the well-studied Northern Hemisphere, aerobiological studies of the Southern Hemisphere have been conducted in isolation and limited to monitoring specific cities for narrow time periods with few attempts made to discern generalised patterns across a country or region (see [13] for an exception). Indeed, many of the existing aerobiological studies located in Australia and New Zealand have been motivated by an interest in the public health burden of pollen sensitization and allergic asthma at major population centres. Aerobiology in the Southern Hemisphere is as complex as that in the Northern Hemisphere, given the variability of abiotic factors and environmental gradients that span the tropics to the temperate zones. The coexistence of very distinct indigenous vegetation, introduced Northern Hemisphere ornamentals, as well as exotic invasive species are additional variables that contribute to the aerobiology of each Southern Hemisphere regional center. Here we compile and analyse an historic atmospheric pollen dataset from 11 cities across Australia and New Zealand and examine the regional variations in pollen content and relative abundance. The geographical distribution of the 11 Australasian sites was broad enough to enable further examination of the role that biogeography plays in regional and urban variations in pollen composition. These data can potentially provide insights into how pollen production and dispersal derived from both native and exotic taxa may respond to changes in climate and urban and peri-urban land use. The influence of these variables and how that may affect allergic diseases is also discussed.

We emphasise that these results were achieved by collating data sets that were originally obtained for other purposes and were often of limited duration and collected using a variety of methods. The combined data set provides impetus for further work and suggests the potential of a more systematic and coordinated pollen monitoring network in Australasia for long-term studies into environmental change and the production of more accurate pollen forecasting systems to help allergy sufferers and their health care providers better manage their conditions. We conclude by considering some important issues that such an initiative could address.

## Materials and Methods

### Sampling Sites

**Figure 1:**
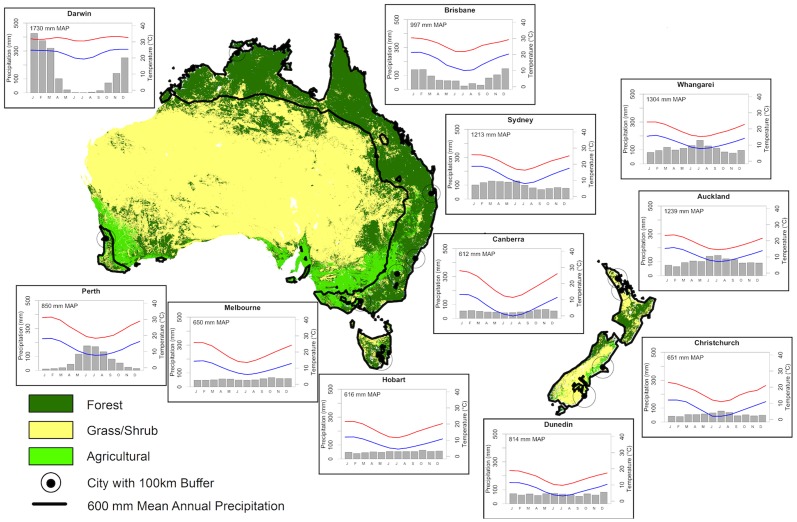
Land cover attribute for Australia and New Zealand with climatological summaries for each major urban centre associated with an aerobiology study. Climate summaries include average monthly precipitation and minimum and maximum temperatures for each urban area (see Table 3 for data sources). Data shown for Whangarei represents the Kaikohe pollen count site.

Aerobiological data have been collected across Australia and New Zealand using a range of methods and counting periods (see Table 1). This paper describes the re-analysis of approved university studies of aerobiological pollen in Australia and New Zealand. All studies have been published (Table 1) with the exception of pollen data from Perth and Canberra, where unpublished data were provided by the investigators at these sites (also authors in this paper FM and SGH). In cities where more than one station is operating the secondary station is not included in the numerical analysis as they overlap and are shorter records than the nearby stations in the same cities. Land cover attributes for Australia and New Zealand with climate summaries for each major urban centre associated with aerobiology studies are shown in Figure 1. Table 1 provides the location details of each aerobiological station including the collection periods of 14 aerobiology stations across 11 urban centres, which have been in operation for at least one season over the last 25 years. In aerobiology, pollen traps are situated well above ground level, both to avoid the complicating influence of local vegetation and anthropogenic disturbance as well as to better sample the regional pollen flux that are dominated by the major anemophilous (wind-dispersed) taxa [19]. This group of wind-pollinated species comprise many of the important allergenic sources [20]. The data presented here were obtained at pollen sampling sites ranging in elevation above ground level that, along with other differences in collection methods described below, are likely to affect the total volume of pollen sampled. Nevertheless the methods used at each location provide estimates of airborne pollen volume (in grains/cm^3^) that can be compared between regions and through time.

**Table 1:** Sites and methodologies employed in aerobiology recording stations across Australia and New Zealand.

Location name	Suburb (site)	Country (State/Territory)	Latitude (S)	Longitude (E)	Elevation (m)	Monitor Height (m)	Start Date	End Date	Duration (days)	Time step	Time Start/End	Monitor type	Magnification	Counting method
Darwin	Casuarina (Charles Darwin University)	A (NT)	12°22′	130°52′	13	14	18/03/2004	15/11/2005	607	24 hrs	0:00	Burkard	400×	4 transects
Darwin	Palmerston	A (NT)	12°28′	130°58′	24	14	1/04/2004	31/03/2005	364	24 hrs	0:00	Burkard	400×	4 transects
Brisbane	Rocklea	A (QLD)	27°29′	153°08′	13	2	7/06/1994	17/05/1999	1805	7 day	unknown	Burkard	250×	entire slide
Perth	Murdoch (Murdoch University)	A (WA)	32°04′	115°50′	30	unknown	1/09/2006	31/12/2006	121	24 hrs*	0:00	Burkard	×400	3 transects
Sydney	Campbelltown (University of Western Sydney)	A (NSW)	34°04′	150°47′	74	10	1/01/1993	31/12/1995	1094	24 hrs	unknown	Burkard	400×	3 transects
Canberra	Acton (Australian National University)	A (ACT)	35°16′	149°7′	569	8	26/09/2007	13/12/2009	809	24 hrs	0:00	Burkard	400×	4 transects
Canberra	Holder	A (ACT)	35°19′	149°2′	570	14	27/8/09, 29/9/10	31/1/10, 26/11/10	210	24 hrs	10am (2009), 4pm (2010)	Burkard	×40 (2009), ×20 (2010)	1 transect
Melbourne	Parkville (Melbourne University)	A (VIC)	37°48′	144°17′		14	1/09/2009	31/12/2011	852	24 hrs	16:00	Burkard	20× with a field of view of 1 mm	1 transect
Hobart	Sandy Bay (University of Tasmania)	A (TAS)	42°54′	147°19′	58	12	6/09/2007	31/12/2010	1212	24 hrs	0:00	Burkard	400×	4 transects
Kaikohe	Kaikohe	NZ	35°24′	173°48′	198	2	15/11/1988	11/02/1989	88	24 hrs	12:00	Rotorod	400×	n/a
Auckland	Grafton (Museum)	NZ	36°51′	174°46′	61	8	28/10/1989	27/04/1990	181	24 hrs	9:00	Rotorod	400×	n/a
Auckland	Onehunga	NZ	36°51′	174°46′	15	3	27/10/1989	30/04/1990	185	24 hrs	9:00	Rotorod	400×	n/a
Christchurch	Christchurch (Canterbury University)	NZ	43°31′	172°35′	14	20	17/11/1988	13/02/1989	88	24 hrs	12:00	Rotorod	400×	n/a
Dunedin	Dunedin	NZ	45°51′	170°30′	0	5	1/10/1992	31/01/1993	122	24 hrs	9:00	Rotorod	400×	n/a

Data derived from unpublished and published data (Newnham *et al*. [13]; Bass and Morgan [14]; Ong *et al*. [15]; Green *et al*. [16]; Stevenson *et al*. [17]; Tng *et al*. [4]; Medek *et al*. [18]). Note that data from secondary stations in Darwin (Palmerston), Canberra (Holder) and Auckland (Grafton Museum) are not included in the numerical analysis in this paper. The raw pollen counts for each site used in this paper have been archived in excel files at the Australian Centre for Ecological Analysis and Synthesis data portal (http://aceas-data.science.uq.edu.au/portal/). Click on the following hyperlinked text to download data from each aerobiology recording station. Darwin, Brisbane, Perth, Sydney, Canberra, Melbourne, Hobart, Kaikohe, Auckland, Christchurch, Dunedin.

Assumptions: All air sampled at 10 l/minute (except NZ rotorod: 1.59×32 mm rods spun at 2400 rev/min for 6 min/hr), and all express concentration as grains/m3 of air.

*Not Saturdays and Sundays.

A; Australia, NZ; New Zealand, NT; Northern Territory, QLD; Queensland, WA; Western Australia, NSW; New South Wales, ACT; Australian Capital Territory, VIC; Victoria, TAS; Tasmania.

### Pollen Data Collection

The most common method of daily pollen counts in Australia is based on the deployment of a seven-day Hirst-type volumetric pollen and spore trap [21] located on structures (mostly rooftops) ranging from between 2–14 m above the ground. In New Zealand, an alternative device, the Intermittent Cycling Rotorod sampler [22] was deployed at all sites, but provides similar volumetric data. The Hirst-type sampler uses a range of adhesive surface compounds including vaseline and 10% paraffin wax in toluene on Melinex™ tape (Burkard Manufacturing Co. Ltd., Rickmansworth, Hertfordshire, UK), silicon-based adhesive (Lanzoni s.r.l., Bologna, Italy) or a 50% saturated Dow Corning high vacuum grease in solvent [23]. The seven-day tapes are then cut into 24-hr segments and mounted on glass slides with a stain such as fuchsine stained Gelvatol [24], Calberla's stain, or 2% Saffranin O in glycerol jelly. Alternatively daily pollen monitoring can be done using a glass microscope slide that is coated and stained as described. Analysis of each 24-hr period is conducted by counting between one and four transects at 400 magnification [25], though at one site the entire slide surface was counted (Brisbane, [26]). Pollen and spore counts were then converted to grains/m^3^ of air and expressed as a daily mean value [21]. Hirst-type pollen and spore traps are known to show an instrumental variation of about 25% [27]. In New Zealand the Intermittent Cycling Rotorod sampler is an impaction collector with a retracting collector rod sampling head [22]. Particles are collected on the leading, greased, edge of two 1.59×32 mm clear polystyrene collector rods spun intermittently by an electric motor at 2400 rev./min. The samplers were set-up to operate for 6 min every hour. Sampling rods were replaced every 24 hours, stained with Calberla's solution, and examined under a transmitted light microscope. Raw pollen counts for each of the pollen/spore types identified were converted to the volumetric index (grains/m^3^ of air) using a standard formula taking into account the sampling period and volume of air sampled [22]. The daily mean concentration of fungal spores, particularly *Alternaria*, was also recorded at some stations (Darwin, Brisbane, Sydney, Canberra, Melbourne and Hobart), though detailed analysis of these data is not presented here. The raw pollen counts for each site used in this paper have been archived in excel files at the Australian Centre for Ecological Analysis and Synthesis data portal (http://aceas-data.science.uq.edu.au/portal/). Links to specific station datasets are provided in Table 1.

### Pollen Identification

Pollen identification was aided by the existing reference collections held by individual analysts or collated into digital or online reference collections (e.g., the Department of Archaeology and Natural History at the Australian National University, http://apsa.anu.edu.au, the CD published by Hjelmroos *et al*. [28]). In a number of these aerobiological surveys, it was not possible to place identifications at a species or genus taxonomic level. In these cases the taxon name reverted to the higher level (i.e., all genus-level identifications were placed into the relevant family) such as the Poaceae and Myrtaceae. The introduced tree taxa are referred to by their generic name (*Alnus, Betula*, *Pinus, Quercus*, *Salix and Ulmus*) as all are represented in Australia and New Zealand by more than one species in the genus and these are not distinguished in the available datasets.

### Pollen Count Analysis

In order to reduce variation in pollen abundance records due to variations in equipment or handling protocols, the datasets were transformed into percentage values based on a total pollen sum (total pollen abundance of a single taxon recorded over the time of study divided by the total pollen abundance of all taxa recorded over the time of study) (Table 2). This data transformation mitigated other sources of variation in abundance between monitoring sites such as location, as coastal cities are exposed to a smaller proportion of land area and hence vegetation sources than inland cities (Figure 1).

**Table 2:** Aerobiologically significant pollen taxa contributing >80% to the total annual atmospheric pollen in urban areas across Australia and New Zealand.

Taxa	Darwin	Brisbane	Perth	Sydney	Canberra	Melbourne	Hobart	Kiakohe	Auckland	Christchurch	Dunedin	Ave (rank)
Arecaceae	26.0											*26.0
*Casuarina*	3.1	6.5		6.2	1.8	0.4	4.3					3.7 (8)
Urticaceae	1.6	1.8		0.7	1.9	0.9			0.1			1.2 (16)
*Acacia*	3.1	0.2		0.3	0.3	0.3	0.5		<0.1			0.7 (18)
Myrtaceae	31.0	3.1	6.0	11.0	5.7	5.6	8.2	<0.1	5.2	3		7.9 (4)
*Chenopodiaceae*	0.3	0.1			0.8	0.2	0.3	<0.1	11.0	6.8		2.4 (12)
Cyperaceae	5.5	1.3		0.3	1.2		0.5			0.03		1.5 (13)
Asteraceae	0.1	0.7	2.5	1.0	0.8	0.4	0.5	0.3	2.1	2		1.0 (17)
Cupressaceae	5.2	9.0	27.5	23.0	22.5	58.0	13.3	0.4	1.0	1.7	28.5	17.3 (2)
*Pinus*	0.1	4.5	31.0	3.0	17.0	1.2	3.3	0.3	0.5	2.4	11.4	6.8 (5)
Poaceae	18.0	71.0	23.0	17.5	15.0	10.8	10.4	84.0	43.7	51.6	25.8	33.7 (1)
*Plantago*		0.2		5.1	3.2	1.8	3.4	11.1	16.0	8.6		6.2 (7)
*Ulmus*				<0.1	3.8	0.4	1.6					1.5 (14)
Oleaceae				20.0	1.8	5.2	5.2		<0.1		7.2	6.6 (6)
*Salix*				0.2	1.7		5.0		<0.1	0.2		1.4 (15)
*Rumex*					4.3	0.1	2.3	3.6	3.5	6.5		3.4 (9)
*Quercus*				0.1	3.1	4.9	1.6	<0.1	0.2	11.2		3.0 (10)
*Alnus*					3.9	1.8	2.0					2.6 (11)
*Betula*					1.7	4.5	16.7		0.6	0.35	27.0	8.5 (3)
*Coprosma*							0.4	<0.1	0.1	1.7		0.6 (19)
Sub-total	93.9	98.3	90.0	88.3	90.5	96.3	79.4	99.8	84.0	96.1	99.9	92.4
Other	6.1	1.7	10.0	11.7	9.5	3.7	20.6	0.2	16	3.9	0.1	7.6

Pollen taxa values are expressed as a percentage of the total pollen count for the record period available in each urban area (*excluded from ranking as only occurs in one urban area). The average and rank of percentage values across all urban areas is given in the right hand column. The category “Other” includes all pollen taxa counted in the total pollen sum and not identified to taxa here.

**Figure 2:**
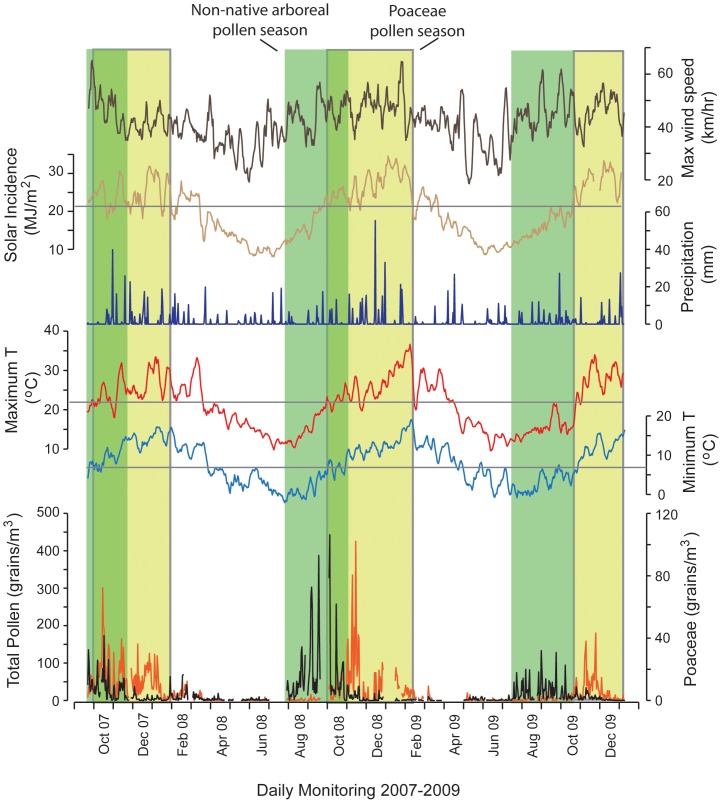
Climate summary and daily airborne pollen (Poaceae and Non-native arboreal taxa) for Canberra (26 Sept 2007–31 Dec 2009). Pollen season is depicted by the shaded columns and defined by 90% of the airborne pollen falling in this time for each year (July 1 to June 30). Climate data from the Australian Bureau of Meteorology.

The pollen season has been defined in many ways by different authors [29]. To date, no standardized approach to identifying the pollen season has been defined in Australia or New Zealand [13], [15]–[17]. In general, the pollen season is said to have four parameters, the start, duration, peak and the end. In this study we adopted the Nilsson and Persson [30] approach, where the ‘start day’ is when the sum of daily pollen concentrations reaches 5% of the total yearly count and the ‘end day’ when the sum reaches 95% of the yearly count. The ‘year’ for all subtropical and temperate sites began on July 1 and ends on June 30. The exception was the tropical site (Darwin), where the ‘year’ begins on January 1 and ends on December 31. The season is thus the period when 90% of the total annual pollen count is collected on the trapping surface (see example given in Figure 2). Here we also point out that the years of sampling varied between certain sites, ranging from 1988 to 2012, with some sites sampled for a single season, a single year, or a small number of years. This factor must be considered when comparing phenological observations such as the timing of pollen season parameters between sites, as pollen season for the same species may vary substantially between years [31].

Similarities between the airborne pollen assemblages recorded within the 11 urban areas were calculated using non-metric multidimensional scaling ordination (nMDS, [32]), a technique that reduces the dimensionality of multivariate data, and graphically represented using the relative dissimilarities amongst (a) urban areas and (b) dominant taxa. The robust nature of the ordination is measured through the numerical stress value. A stress less than two corresponds to a good ordination and useful two-dimensional picture of sample similarity [32]. The nMDS outputs show vector positions for each variable within the environmental space. The length of the vectors correspond to the square-root of the r^2^ values, so weak predictors have a shorter length than strong predictors. High r^2^ values indicate a vector that is strongly associated with site/species variation in the ordination space. Low r^2^ values would indicate a vector that doesn't really explain much in the ordination space. Environmental vectors representing three meteorological variables (Mean annual maximum and minimum temperature and mean annual precipitation) and the proportion of four broad land cover types in a 100 km radius around each urban area were fitted to the species ordination using the envfit function of the vegan package in R [33], with random permutations of environmental variables performed in order to assess their significance. We make the assumption that the proportion of land cover types found in a 100 km radius around pollen sampling stations is representative of the total potential pollen dispersal area. However, it must be acknowledged that little is known of the pollen dispersal characteristics of Australasian pollen types and that further research is required to understand the influence that meteorological factors such as wind strength and distance from source vegetation might have on pollen concentrations in the atmosphere.

## Results

### Pollen Ranking Among Urban Areas

**Figure 3:**
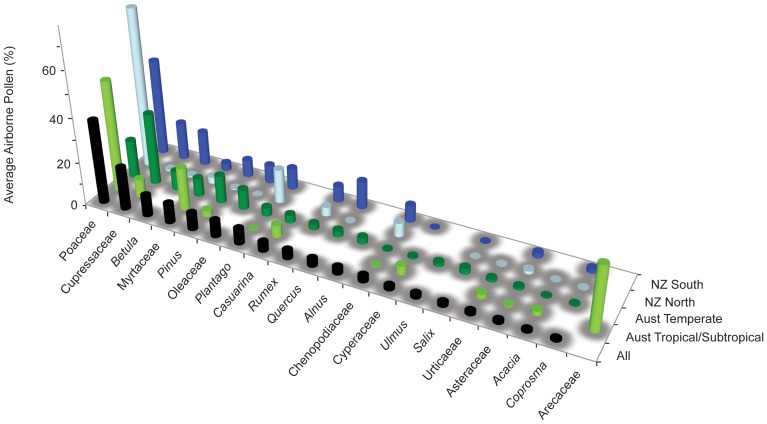
Ranking for top 20 pollen taxa based on average percentage representation of airborne pollen areas across all urban areas (black bars). These urban areas are then grouped into regional biogeographic zones (blank space = taxa not recorded). Australian Tropical/Subtropical = Darwin, Brisbane; Australian Temperate = Perth, Sydney, Canberra, Melbourne, Hobart; NZ North = Kaikohe, Auckland; NZ South = Christchurch, Dunedin.

The airborne pollen data were ranked by percentage representation, with the top 20 pollen taxa making up >80% of the total pollen collected across 11 cities in Australia and New Zealand (Table 2 and Figure 3). The dataset incorporates taxa of high relative percentage representation that commonly occur in two or more urban areas (Arecaceae is an exception because of the high values recorded in Darwin). The most significant taxa across all sites are Poaceae and Cupressaceae, making up over 50% of the total airborne pollen in urban environments throughout the year. These are followed by *Betula*, the trees and shrubs in Myrtaceae, *Pinus*, Oleaceae, *Casuarina*, and the important herbaceous taxa such as *Plantago* and *Rumex*. Differences between the pollen taxa rankings (highest to lowest percentage representation) across the broad biogeographic regions of Australia (tropics to temperate: Australian Tropical/Subtropical = Darwin, Brisbane; Australian Temperate = Perth, Sydney, Canberra, Melbourne, Hobart) and New Zealand (North and South Island: NZ North = Kaikohe, Auckland; NZ South = Christchurch, Dunedin), reflect the strong climate controls on plant distributions, particularly those associated with Northern Hemisphere introductions such as *Betula*, *Quercus*, *Alnus* and *Ulmus* that contribute high levels of pollen into the atmosphere of the southern temperate cities.

### Biogeographic Patterns

**Figure 4:**
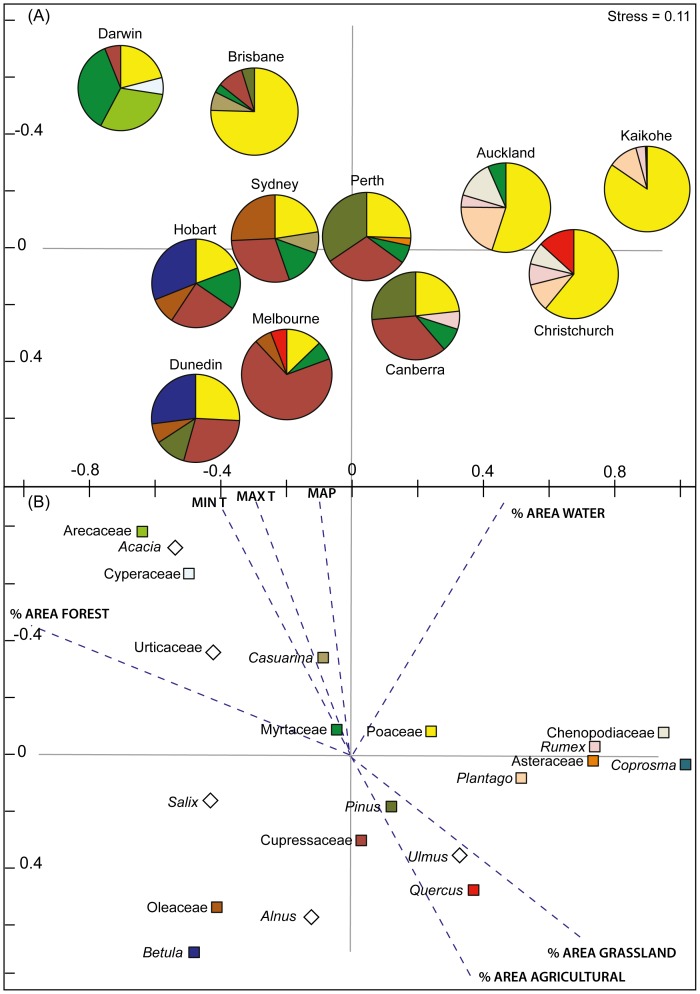
Non-metric multidimensional scaling ordination (nMDS) of the major pollen taxa in Australia and New Zealand. (**A**) Distribution of urban areas using a matrix of percentage representation of major pollen taxa (see Table 2 and using top eight pollen taxa at each site). The pie charts depict the relative contribution of the most abundant pollen taxa in each urban area. (**B**) Distribution of pollen taxa contributing to the differentiation of aerobiology of each urban area Note: coloured squares associated with each taxa depicted in (B) match the pie chart colours shown in (A), with diamonds showing taxa with a low percentage representation. The vectors (dotted lines) for the environmental variables show positions for each variable within the environmental space. Longer vectors (higher r^2^ values, see Table 3) indicate a stronger association of the environmental variable with site/species variation in the ordination space.

The two dimensional ordination had a low stress level (stress = 0.11) indicating it was a robust graphical representation of the similarity matrix among the cities and the dominant taxa. The ordination displays a gradation in vegetation formation (and land cover) across the set of urban areas (Figure 4A). The x-axis separates the tree dominant urban landscapes (negative values associated with pie charts dominated by tree taxa) from the grassland dominated urban landscapes (positive values associated with pie charts dominated by herbaceous taxa). The y-axis is associated with latitudinal position of Australian and New Zealand sites with positive values corresponding to lower latitude urban areas and negative values corresponding with higher latitude urban areas. The most significant environmental variable explaining the difference between airborne pollen in each urban area was minimum annual temperature (MinT, r^2^ = 0.77, P = 0.002), which is closely aligned to the latitudinal transect along which the urban areas lie (Table 3). The second environmental factor that showed a significant correlation with the airborne pollen was mean annual precipitation (MAP, r^2^ = 0.66, P = 0.01). Increased grass pollen dominance is clearly apparent in Figure 4, particularly in the savanna dominated tropics (Darwin), dry sclerophyll subtropical forests (Brisbane) and pastoral dominated temperate landscapes of New Zealand, where associated ruderal taxa such as *Rumex*, *Plantago*, Asteraceae and Chenopodiaceae are also prominent. The contribution of grass pollen was reduced in the southern temperate cities, where other woody taxa such as Cupressaceae (including both native and introduced species) and the introduced Northern Hemisphere trees (*Betula*, *Alnus*, *Ulmus*, and *Quercus*) become significant contributors.

**Table 3:** Land cover attributes within a 100

Urban Area	%Area Water	%Area Agricultural	%Area Grass	%Area Forest	MaxT (°C)	MinT (°C)	MAP (mm)
**Darwin**	45.5	0.1	0.2	54.3	33.3	19.3	1730
**Brisbane**	34.9	1.5	0.1	10.1	30.3	10.0	997
**Perth**	38.1	31.6	3.5	26.8	31.4	7.7	850
**Sydney**	51.1	4.2	0.3	44.3	25.9	8.0	1213
**Canberra**	2.2	30.2	5.1	62.5	28.0	−0.1	612
**Melbourne**	19.0	27.6	14.3	39.1	25.9	6.0	650
**Hobart**	40.2	6.3	7.3	46.2	21.6	4.5	616
**Kaikohe**	65.1	0.6	22.2	12.2	24.3	7.8	1304
**Auckland**	62.9	2.2	24.8	10.1	23.7	7.1	1239
**Christchurch**	57.9	20.5	18	3.6	23.0	2.0	651
**Dunedin**	62.4	6.1	31.1	0.4	18.9	3.2	814
**nMDS Axis 1**	0.48	0.36	0.72	−0.89	−0.29	−0.40	−0.11
**nMDS Axis 2**	−0.88	0.93	0.70	−0.45	−0.96	−0.92	−0.99
**r^2^**	0.14	0.39	0.33	0.34	0.38	**0.77**	**0.66**
**Pr(>r)**	0.568	0.164	0.205	0.195	0.154	**0.002**	**0.013**

The significant relationships (p<0.05) between environmental variables and airborne pollen are indicated in bold (T = temperature, MAP = Mean Annual Precipitation). Meteorological data derived from Worldclim [34] and land cover data from Giri *et al*. [35].

### Pollen Seasons

**Figure 5:**
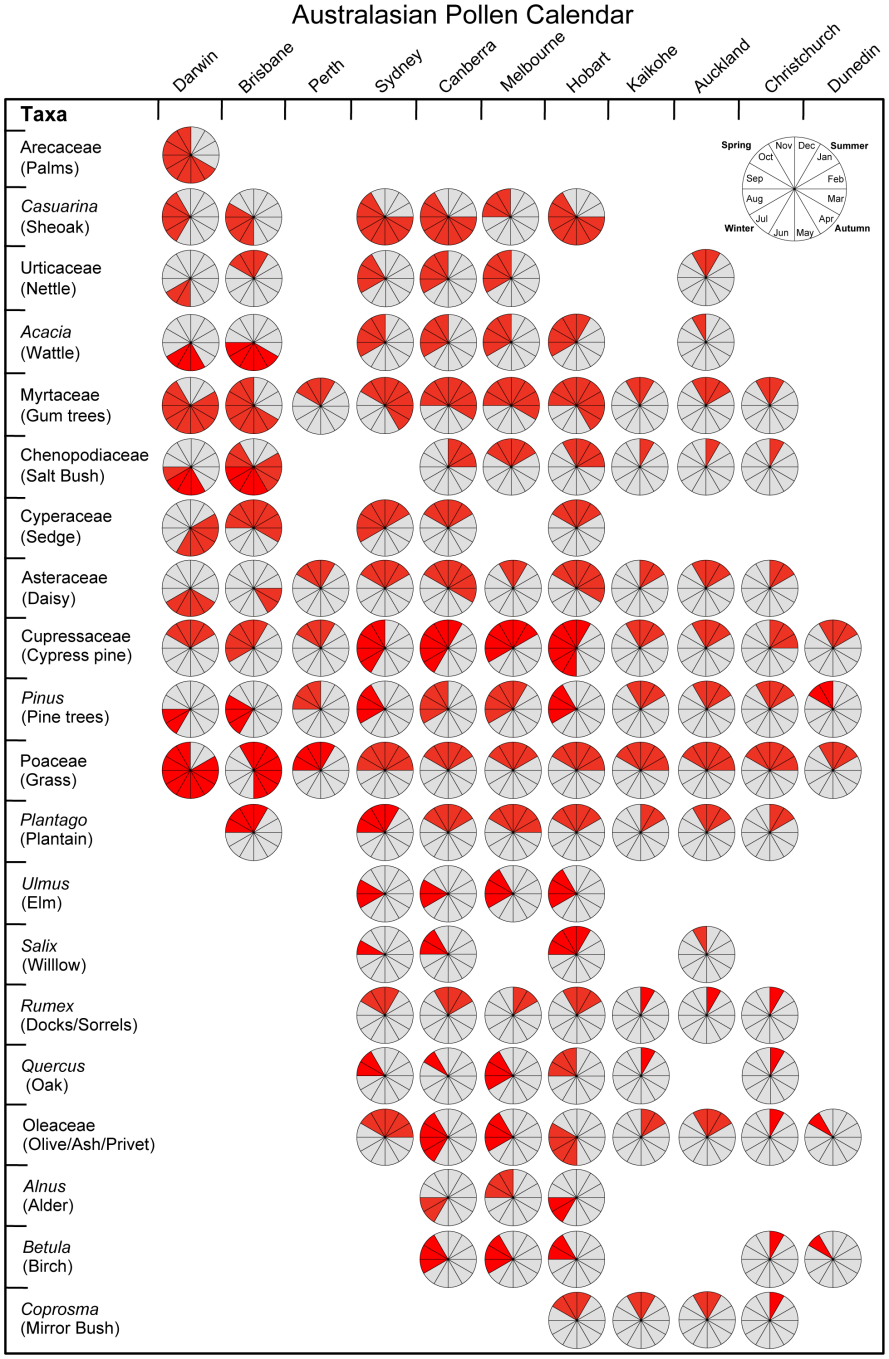
Pollen calendar for aerobiologically significant pollen taxa (contributing 80%+ to the annual atmospheric pollen) in Australian and New Zealand urban areas. The pie charts are divided into monthly segments with the red shade depicting the pollen season for each taxa to the nearest month in a given urban area. The pollen season for each taxon is determined using the period encompassing 90% of the annual pollen rain (see Figure 2).

Figure 5 illustrates the Australasian pollen calendar for the top 20 taxa across 11 urban areas. The matrix is organised according to geographical distribution from tropical to temperate for both urban areas and pollen taxa. The clear shifts from long pollen seasons in the tropics to shorter periods in the temperate regions reflects the strong control of solar radiation incidence on pollen production during spring and summer months in the southern urban areas [36]. There is also a discernible shift in the initiation and length of pollen season towards higher latitude. For example, Poaceae has a long flowering season in the northern cities of Darwin and Brisbane (across the dry season and into the wet season as different grass species flower through the year), compared to the progressively shorter and later initiation of the pollen season in the southern cities.

The effect of temperature and solar incidence on the beginning and end of the pollen season in the temperate zone is illustrated with an example from Canberra in Figure 2, where the relationship between airborne pollen and key climate variables for Poaceae and total non-native tree species (Northern Hemisphere) is shown. While this is simply a visual comparison, previous studies from Melbourne [15], [37], Brisbane [26] and New Zealand [38] have also documented the significant influence of temperature and precipitation on the commencement and duration of the grass pollen season. The lack of consistent pollen season in some taxa such as Oleaceae may be a function of the multiple species from different genera being incorporated into this taxon (including *Olea* and *Fraxinus*), reflecting a significant limitation imposed by the inability to resolve pollen to the species level.

## Discussion

The history of aerobiology research in Australia and New Zealand, like the rest of the world, can be characterised by its focus on local site (urban area) issues. Unlike other continents however, where considerable effort has been applied to standardise methodologies, teams working in Australia and New Zealand have tended to use a variety of collection and counting methodologies with little or no co-ordination between aerobiology stations in different urban areas. Despite the idiosyncrasies of the data, this study supports a basic principle that underpins palynology – that airborne pollen is a sensitive proxy of the current climate and flora of a region. Our results indicate that this principle applies as much to the introduced flora as to indigenous vegetation, despite concerted human effort to manipulate the former.

We found that Australian and New Zealand urban areas from a similar climate zones have similar pollen spectra but important differences occur due to surrounding land use and the establishment of non-native plants. Likewise, in similar climate zones, pollen season of each taxon is similar but not the same. For example, urban areas are often surrounded by agricultural landscapes that have a diversity of pollen predominantly represented by grass pollen and characterized by short seasons (Figure 5) compared to urban areas where there is more surrounding forest cover (e.g. Sydney and Hobart). Changes in land use on urban boundaries also have the potential to affect the types of airborne pollen inside the urban area [39]. The predominance of exotic and invasive tree and shrub species (and certainly non-native grasses, albeit these cannot be taxonomically resolved) highlights the profound changes that have occurred following European colonisation of Australia and New Zealand. For instance, in the urban areas of southern Australia and New Zealand, the widely planted birches are particularly dominant.

Temperature and rainfall are known to be primary controls on the daily distribution of airborne pollen in Australia and New Zealand, however, other climate variables that may influence the dissemination of these allergenic pollen types in the atmosphere are not well understood. Other variables such as the El Niño-Southern Oscillation (ENSO) have not been investigated, even though this large-scale ocean-atmosphere anomaly has been shown to modify climatic patterns, leading to droughts and floods, which have local and regional implications on the biosphere as well as vector borne diseases. A similar climate oscillation in western Europe, the North Atlantic Oscillation, has been linked with seasonal variation in grass pollen (e.g. [40]). ENSO and other short term climate oscillations such as the Southern Annular Mode and Indian Ocean Dipole may be important variables that could account for interseasonal differences observed in airborne pollen counts and seasonal starting dates in Australasia. The broad-scale patterns in the distribution, abundance and season of pollen amongst Australasian urban areas provide some clues as to potential changes in aerobiology due to climate change. Evidence from Europe and North America demonstrates that climate change has already increased the abundance and seasonal duration of allergenic pollens such as birch and ragweed and possibly increased concentrations of allergenic compounds [9], [12], [41]. Changes in the burden of allergenic disease related to changing climate have also been demonstrated [41], [42].

In marked contrast to developed countries of the northern hemisphere [43], the potential allergenic impacts of endemic southern hemisphere plants are poorly characterized despite many being identified as important allergens. Given the widespread population exposure to known allergenic plants we endorse the recommendation of the 2007 study of the economic impact of allergic diseases in Australia by Access Economics that *‘studies of the aerobiology and clinical significance of potential native Australian triggers of respiratory allergic disease should be made a priority’* [44]. Possible triggers include both native and non-native taxa. Native genera include common woody plants (*Eucalyptus*, *Melaleuca*, *Callistemon* and *Acacia* [45]–[51]) and grasses (*Sorghum*, *Sarga* and *Andropogon* [52]–[54]). Non-native plants include a number of taxa known to be allergenic. For example, birch is an important allergenic pollen in Scandinavia and a notable allergen throughout north-central Europe [6], [55] and it has been identified as an important allergenic pollen type in southern Australian urban areas [56]. Similarly, the non-native gamba grass (*Andropogon gayensis*) is thought to have lengthened the pollen season and increased the community burden of allergic rhinitis in Darwin [57].

Exotic allergenic plants have the potential to cause profound public health impacts if their ranges were to expand and their population to increase. For example, pollen produced by olive trees (*Olea europaea*) is the leading cause of seasonal allergic diseases in some regions of southern Europe [58], and this species is expanding its range across mediterranean climate zones in Australia [59] adding a new allergic pollen in areas where there are already allergic pollen in the atmosphere. If Ragweed species (*Ambrosia* spp.) were to expand their range and abundance they would add a new source of allergenic into autumn [60]–[62], a season currently with very few types of pollen (see Figure 5). Because allergic sensitisation to multiple plant allergens is common [63], our results show that the juxtaposition of non-native tree pollen such as birch in early-mid spring with Poaceae pollen in mid-late spring and summer could result in a lengthened period of risk for people allergic to pollens (cf. Figure 2).

## Conclusions

Our study highlights the need to monitor changes to the aerobiology and provides a framework for targeting the most important taxa in terms of abundance and allergenic effects for each urban area. Establishing systematic regionally-based monitoring of airborne pollen will enable Australian and New Zealanders to better understand the high taxonomic diversity and seasonal variability of allergenic pollens. This will redress the paucity of research on the clinical and public health impacts and treatments for common endemic allergenic species such as *Eucalyptus* and *Sorghum*. Monitoring the increasing abundance of allergenic exotic species populations that can move from a dormant ‘sleeper’ populations to aggressive expansion phases, Ragweed (*Ambrosia* spp.) being a example, will also be a priority. Understanding the impacts that climate change will have on the phenological cycles and range of allergenic species into the future will be a critical step in the advancement of aerobiology studies in the Australasian region [9].
